# Fertility preservation and fulfillment of parenthood after treatment of hematological malignancies: results from the ‘Aftercare in Blood Cancer Survivors’ (ABC) study

**DOI:** 10.1007/s10147-020-01639-4

**Published:** 2020-03-05

**Authors:** Christine Schmitz, Julia Baum, Hildegard Lax, Nils Lehmann, Tanja Gromke, Dietrich W. Beelen, K.-H. Jöckel, Ulrich Dührsen

**Affiliations:** 1 Department of Hematology, West German Cancer Center, University Hospital Essen, University of Duisburg-Essen, Hufelandstraße 55, 45147 Essen, Germany; 2 Institute for Medical Informatics, Biometry and Epidemiology, University Hospital Essen, University of Duisburg-Essen, Essen, Germany; 3 Department of Bone Marrow Transplantation, West German Cancer Center, University Hospital Essen, University of Duisburg-Essen, Essen, Germany

**Keywords:** Posttreatment parenthood, Hematological malignancy, Fertility preservation, Treatment-related infertility

## Abstract

**Purpose:**

Treatment of hematological malignancies carries the risk of lasting sterility. We aimed to identify fertility-related unmet needs.

**Methods:**

The ‘Aftercare in Blood Cancer Survivors’ study is a cohort study of hematological patients who were in treatment-free remission for ≥ 3 years or stable under continuous oral medication. Female patients age 18–45 years and male patients age 18–65 years without a history of pre-treatment infertility were asked to answer a structured questionnaire including questions addressing fertility issues. Multivariable analyses were performed to detect risk factors.

**Results:**

Of 1562 study participants, 1031 met the inclusion criteria for the fertility sub-study. A high proportion of patients (72.4%) received information about the risk of losing fertility, but only a minority (15%) took steps to preserve it. Female and older patients were less likely to be informed. A post-treatment wish for parenthood was expressed by 19.3% of patients. It was strongly associated with childlessness at time of diagnosis and could be fulfilled by 29.4%. Fulfillment of desired parenthood increased with increasing time from diagnosis and was low after allogeneic transplantation.

**Conclusions:**

Female and older hematological patients are less likely to be informed about fertility-related issues than other patients. With societal changes towards first parenthood at higher age, the proportion of patients desiring a child after treatment is likely to increase. Fulfillment of desired parenthood remains challenging, especially after allogeneic transplantation.

**Implications for cancer survivors:**

In patients likely to express a wish for post-treatment parenthood, fertility-related issues should routinely be addressed before gonadotoxic treatment is started.

**Electronic supplementary material:**

The online version of this article (10.1007/s10147-020-01639-4) contains supplementary material, which is available to authorized users.

## Introduction

Overall survival in patients with hematological malignancies has markedly improved. In some types of aggressive disease, such as lymphoma, cure rates are high, and in indolent malignancies, long-lasting remissions can be achieved [1]. Due to a growing number of long-term survivors, patient care is increasingly being focused on the optimal management of treatment side effects.

Lasting infertility is one of the most crucial side effects of chemotherapy as it can sustainably affect a cancer survivor’s concept of life. The majority of young cancer survivors express a desire for a child [2–5], and fear of persisting infertility causes distress and limits the quality of life [6]. Fertility preservation, such as cryopreservation of sperm, oocytes, or ovarian tissue, is a promising approach to enable post-treatment parenthood [7–9]. Moreover, the use of fertility preservation was shown to help patients cope with their diagnosis of cancer [10]. However, fertility preservation is often insufficiently discussed [4, 11, 12], and referral rates to fertility institutes are low [4, 12–14]. On the part of the treating oncologist, one of the main reasons accounting for this situation is fear to lose time when treatment is urgent [15–17]. Only a few studies focus on the patterns of fertility preservation in hematological patients [13, 18].

The present report is a sub-study of the ‘Aftercare in Blood Cancer Survivors’ (ABC) study which analyzed current patterns of follow-up in patients with hematological malignancies. The core study focused on medical events, quality of life, and costs during follow-up, and the results will be presented separately. Here, we report on receipt of pre-treatment information about the risk of treatment-induced infertility, use of fertility preservation as well as wish and fulfillment of post-treatment parenthood, as reported by the patients in the retrospective part of the ABC study. We also identified patient- and disease-specific factors associated with these issues.

## Methods

### Design

The ABC study is an observational cohort study designed to analyze the current practice of follow-up care in patients with hematological malignancies. It included patients and their treating physicians and recorded events occurring during follow-up from 2014 to 2017. In the retrospective part of the study, patients reported on medical, psychological and social aspects of treatment and follow-up using a 118-item questionnaire. In the second part, medical events, quality of life, and costs were recorded prospectively by involving the physicians providing follow-up care. The fertility sub-study was part of the retrospective investigation.

Patients’ ≥ 18 years with a hematological malignancy or premalignant hematological disorder presenting between 1998 and 2010 for evaluation, therapy or follow-up at the West German Cancer Center in Essen, Germany were eligible. Their disease had to be untreated or in treatment-free remission for at least 3 years. Patients with stable myeloproliferative neoplasms under continuous medication were also included. Questionnaires were sent out to the patients between April and September 2014. With regard to fertility issues, we restricted our analysis to patients who underwent cancer-specific treatment. Women > 45 years and men > 65 years as well as patients with a history of infertility at the time of diagnosis were excluded. The study was approved by the Ethics Committee of the University of Duisburg-Essen under reference no. 14-5692-BO.

### Procedures

The participants were asked to answer a structured questionnaire developed by the investigators. It was subdivided into sections addressing aspects of follow-up care, general medical health issues, and psychosocial well-being. It included eight questions with additional free text options regarding the information received about the risk of therapy-induced infertility, access to and use of fertility preservation, desire and fulfillment of parenthood, need of assisted reproduction techniques, and children born before and after treatment. The questions regarding fertility are listed in the Online Appendix. Information on sociodemographic background, cancer-specific procedures, parenthood at time of diagnosis or marital status was collected using self-reports. Medical reports were used to check for correctness.

### Statistical analysis

Continuous variables are given as means and frequencies as counts (%). The study included four separate analyses. Being informed about the risk of infertility (1) entered multiple logistic regression as endpoint, with patient characteristics and diagnosis as independent parameters. Using fertility preservation measures (2) was modeled similarly. Logistic models were also calculated for the wish to have children (3), and for the fulfilled wish (4). In models (3, 4) the therapy received was also taken into account. Results are given as odds ratios with 95% confidence intervals (95% CI). In (1–3) calculations were performed with full multiple adjustments. Due to the lower number of endpoints in (4), adjustments were made only within diagnoses and therapies.

## Results

### Patients’ characteristics

**Fig. 1:**
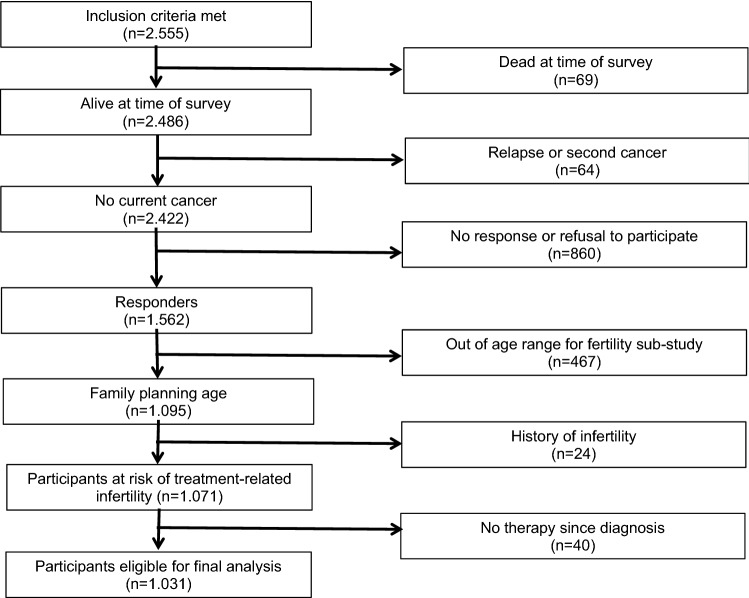
Flow chart of patients

Based on information extracted from the medical files of the West German Cancer Center, 2555 patients were identified to be eligible to participate in the study. After exclusion of recently relapsed or dead patients and patients not responding to the letter or unwilling to participate, 1562 patients were enrolled (Fig. 1). After further exclusion of patients beyond the selected age limits, with a history of infertility, or without treatment since diagnosis, the cohort of the fertility sub-study encompassed a total of 1031 patients (male 68.3%, female 31.7%). The vast majority had received treatment between 1989 and 2010. The median follow-up was 11.5 years (range 3.0–40.8). Patients’ characteristics are detailed in Table 1.

**Table 1:** Patients’ characteristics

Characteristic	Male *n* = 704 (%)	Female *n* = 327 (%)	Total *n* = 1031 (%)
Age at diagnosis			
Mean (years)	42.4	34.1	39.4
18–29 years	130 (18.5)	114 (34.9)	244 (23.7)
30–44 years	262 (37.2)	213 (65.1)	475 (46.1)
45–65 years	312 (44.3)	–	312 (30.3)
Partnership at diagnosis			
Yes	556 (79.0)	247 (75.5)	803 (77.9)
No	130 (18.5)	78 (23.9)	208 (20.2)
Missing information	18 (2.5)	2 (0.6)	20 (1.9)
Children at diagnosis			
No	200 (28.4)	145 (44.3)	345 (33.5)
≥ 1 child	483 (68.6)	172 (52.6)	655 (63.5)
Missing information	21 (3.0)	10 (3.1)	31 (3.0)
Year of diagnosis			
< 1989	23 (3.3)	12 (3.7)	35 (3.4)
1989–2005	453 (64.4)	232 (71.0)	685 (66.4)
> 2005	228 (32.4)	83 (25.4)	311 (30.2)
Diagnosis			
MGUS	1 (0.1)	0 (0.0)	1 (0.1)
Multiple myeloma	24 (3.4)	2 (0.6)	26 (2.5)
Indolent non-Hodgkin lymphoma	108 (15.3)	27 (8.3)	135 (13.0)
Myeloproliferative neoplasm	167 (23.7)	92 (28.1)	259 (25.1)
Myelodysplastic syndrome	19 (2.7)	1 (0.3)	20 (1.9)
Aggressive non-Hodgkin or Hodgkin’s lymphoma	246 (35.0)	116 (35.5)	362 (35.1)
Acute leukemia	139 (19.8)	89 (27.2)	228 (22.1)
Therapy^a^			
(Immuno)Chemotherapy	483 (68.6)	212 (64.8)	695 (67.4)
Autologous SCT	92 (13.1)	34 (10.4)	126 (12.2)
Allogeneic SCT	283 (40.2)	157 (48.0)	440 (42.7)
Others	36 (5.1)	12 (3.7)	48 (4.7)

*MGUS* monoclonal gammopathy of undetermined significance, *SCT* stem cell transplantation

^a^Numbers exceed 100% due to multiple therapy regimens

### Receipt of medical information about the risk of infertility

Among all patients included in the fertility sub-study, 72.4% (male 69.9%, female 77.6%) reported that the risk of infertility had been discussed before the start of treatment. The proportion of patients thus informed declined significantly with rising age (*p* < 0.0001). In patients age 18–29 years, 83.6% of male and 83.9% of female patients reported to have been informed about the risk of infertility, while in patients age 30–44 years, the rate declined to 82.2% in male and 74.3% in female patients. Only 53.2% of men age 45–65 years received information about the risk of infertility.

Multivariable analysis was performed and revealed that higher age (*p* < 0.0001), earlier calendar year of diagnosis (*p* < 0.0001), and female sex (*p* = 0.046) were associated with a lack of information about the risk of therapy-related infertility (Table 2).

**Table 2:** Multivariable analyses for pre-treatment discussion about therapy-related risk of infertility and use of fertility preservation

	Not being informed about risk of infertility		Use of fertility preservation	
	Odds ratio	95% CI	Odds ratio	95% CI
Female sex	1.46	1.01–2.13	0.44	0.27–0.71
Age^a^	1.52	1.37–1.69	0.59	0.50–0.70
Calendar year of diagnosis^a^	0.75	0.65–0.86	1.69	1.38–2.07
≥ 1 child at time of diagnosis	0.94	0.62–1.41	1.17	0.67–2.06
Completed family planning	0.60	0.37–0.99	0.12	0.06–0.25
Disease				
Acute leukemia (reference)	1.0	n.a	1.0	n.a
Multiple myeloma	2.76	1.10–6.91	9.57	1.88–48.75
Indolent non-Hodgkin lymphoma	2.34	1.39–3.97	2.73	0.99–7.49
Myeloproliferative neoplasm	1.27	0.79–2.03	1.62	0.80–3.29
Aggressive non-Hodgkin or Hodgkin’s lymphoma	1.32	0.85–2.05	2.53	1.42–4.52

*CI* confidence interval, *n.a*. not applicable

^a^Per 5 years

### Fertility preservation

Fertility preservation was used by 15.0% of patients (male 14.6%, female 16.0%). It was strongly correlated with age (*p* < 0.0001). In detail, 41.3% of patients younger than 30 years used fertility preservation (male 48.0%, female 33.3%). The proportion significantly decreased with rising age (*p* < 0.001). Among patients age 30–44 years, only 11.2% underwent fertility preservation (male 14.7%, female 6.8%), while none of the men above 45 years underwent cryopreservation of sperm.

On multivariable analysis, female sex (*p* = 0.0007), age (*p* < 0.0001), calendar year of diagnosis (*p* < 0.0001), and completed family planning (*p* < 0.0001) were associated with less frequent use of fertility preservation. With regard to the type of hematological malignancy, patients diagnosed with aggressive non-Hodgkin or Hodgkin’s lymphoma (*p* = 0.0017) or multiple myeloma (*p* = 0.0031) underwent fertility preservation more often than patients suffering from acute leukemia (Table 2).

### Desire for and fulfillment of parenthood after treatment

At the time of diagnosis, the majority of patients (65.7%) had already completed family planning. In patients below 45 years, this was true for 52.2% (male 50.3%, female 54.5%). A desire for a child after the end of treatment was reported by 19.3% of patients (male 17.8%, female 22.3%). Again, we found a strong association with age, as parenthood was desired by 51.2% of patients younger than 30 years (male 57.8%, female 45.1%), but by only 13.9% of patients age 30–44 years (male 17.1%, female 10.0%), and 1.3% of men age 45–65 years (*p* < 0.0001).

Notably, patients that were childless at the time of diagnosis were more likely to express a desire for a child after successful treatment (*p* = 0.012) than patients that already had a child, and patients who preserved fertility before the start of treatment were also more likely to develop a wish for post-treatment parenthood than patients forgoing fertility preservation (*p* < 0.0001; Table 3).

**Table 3:** Multivariable analysis for post-treatment wish for parenthood and univariable analysis for fulfillment of desired parenthood

	Wish for post-treatment parenthood		Fulfillment of parenthood	
	Odds ratio	95% CI	Odds ratio	95% CI
Female sex	0.69	0.45–1.05	0.82	0.42–1.62
Age^a^	0.60	0.52–0.69	0.92	0.72–1.18
Calendar year of diagnosis^a^	1.97	0.16–23.60	0.75	0.60–0.95
Time interval since last treatment^a^	2.12	0.18–25.66	1.34	1.06–1.69
Use of fertility preservation	2.84	1.75–4.61	1.12	0.58–2.16
≥ 1 child at time of diagnosis	0.54	0.33–0.88	3.1	1.54–6.22
Disease				
Acute leukemia (reference)	1.0	n.a	1.0	n.a
Multiple myeloma	0.34	0.03–3.95	–	–
Indolent non-Hodgkin lymphoma	0.61	0.22–1.70	5.13	1.06–24.87
Myeloproliferative neoplasm	0.87	0.48–1.58	0.62	0.17–2.25
Aggressive non-Hodgkin or Hodgkin’s lymphoma	0.83	0.40–1.72	4.39	1.83–10.58
Therapy				
Chemotherapy/exclusive (reference)	1.0	n.a	1.0	n.a
Chemotherapy/combined	1.38	0.86–2.22	0.81	0.35–1.89
Autologous stem cell transplantation	1.53	0.77–3.05	1.52	0.56–4.17
Allogeneic stem cell transplantation	0.86	0.45–1.63	0.18	0.08–0.41
Other	1.07	0.59–1.93	0.84	0.32–2.20

*CI* confidence interval, *n.a*. not applicable

^a^Per 5 years

A wish for parenthood could be fulfilled by 29.4% of patients desiring a child (male 31.1%, female 26.4%), in most cases (78.5%) without medical assistance. Twenty-two men who decided to preserve fertility fathered a child after the end of treatment. Half of them (*n* = 11) reported to have used their stored sperm. The number of patients that fulfilled their desire for parenthood was too low for multivariable analysis. In univariate analysis, fulfillment of desired parenthood was more likely in patients that were already parents at the time of diagnosis (*p* = 0.0015), and in patients with a long interval between treatment and survey (*p* = 0.013). Compared to patients treated with chemotherapy only, patients undergoing allogeneic stem cell transplantation were less likely to fulfill a desire for parenthood (*p* < 0.0001).

## Discussion

Support for post-treatment parenthood should begin before the start of cancer treatment. Patients must be informed about the risk of losing their fertility and the measures that can be taken to preserve it.

In our study, 72.4% of patients reported to have been informed about the risk of infertility. Compared to other studies [13, 19] the rate of informed patients was high. The improvements observed over time are encouraging and suggest rising awareness of hematologists for therapy-induced infertility. At least in part, this may be the result of guidelines published by expert associations, such as the European Society of Medical Oncology and the American Society of Clinical Oncology [20–23].

In contrast to other studies [13, 19], only a minority of patients took steps to preserve fertility. Besides age, we found gender-specific differences to the disadvantage of women, relating both to the receipt of information about the risk of therapy-related infertility and the use of fertility preservation. This observation is in line with findings from other studies [24–26].

Both health care professionals and patients are actively involved in the decision-making process of using fertility preservation. Health care professionals provide information and offer options to preserve fertility, while the patients decide whether they want to make use of the information provided. An important issue not addressed in our study is the question whether the costs were covered by the insurance companies or the patients themselves. Reasons for low rates of fertility preservation may, therefore, be oncologist-related, patient-related or both.

Studies focusing on the behavior of health care professionals concerning fertility issues in cancer patients revealed that oncologists preselect patients on the basis of disease- and patient-specific factors. An urgent need for treatment was one of the main reasons for neglecting fertility-related discussions [15, 16], especially in women [17]. Hematological malignancies often require immediate treatment. Thus, fear of jeopardizing the result of therapy by delaying it in favor of fertility preservation may be one explanation for our findings. This may also explain the differences observed between male and female patients. For men, cryopreservation of sperm is the method of choice, a procedure that is widely available and can be accomplished within a few days. In contrast, fertility preservation in women is more complex. Cryopreservation of embryos or unfertilized oocytes are favored procedures, while suppression of ovarian function by gonadotropin-releasing hormone analogues has shown conflicting results [21]. Harvesting oocytes, however, is a time-consuming process, requiring weeks or months. Cryopreservation of ovarian tissue appears better suited, as it can be done within a few days. In particular, women requiring rapid induction of therapy may benefit from this procedure. However, most hematological malignancies including acute leukemia carry a risk of involving the ovaries [27, 28], which limits the use of ovarian tissue cryopreservation in those who most urgently need it.

Other reasons for not undergoing fertility preservation include unavailability of fertility preservation services and high costs of the procedure that may have to be covered by the patients themselves [29, 30]. As discussed by others, not being able to pay for fertility preservation may embarrass patients, who then deny having been referred to a fertility institute. As a consequence, referral rates are reported to have been lower than they actually were [13].

More than half of our patients reported to have completed family planning by the time the hematological malignancy was diagnosed. A desire for a child after the end of treatment was expressed by only 19.3%. An association of a desire for post-treatment parenthood with young age and childlessness at the time of diagnosis has been noticed before [18, 31]. Not surprisingly, patients who decided to preserve fertility were more likely to express a desire for a child than patients not using fertility preservation. Parental status did not affect the choice to preserve fertility. This implies that patients who are already parents at the time the hematological malignancy is diagnosed refrain from planning to increase the size of their family after completion of therapy. The results of several studies are in line with this observation. While, at the time of diagnosis, patients that already had a child expressed a stronger desire for further parenthood than patients without a child, the opposite was true after the end of treatment [19]. In Hodgkin’s lymphoma, patients that already had a child at the time of diagnosis had lower post-treatment birth rates than patients without a child [32].

Unexpectedly, the desire for a child could only be fulfilled by 30% of the patients. Compared to previous studies [18, 32, 33], the rate found in our study is low. This is likely to be related to the fact that 40% of our patients underwent allogeneic stem cell transplantation, which is known to cause lasting sterility in a high proportion of patients [34–36]. However, an unfulfilled desire for parenthood may not only originate from therapy-induced infertility. Our observation that pre- and post-treatment parenthood were positively correlated, suggests that an unfulfilled desire for a child may also be due to infertility unrelated to chemotherapy. Moreover, childlessness can be caused by infertility of the sexual partner, a possibility that was not investigated in the present study. Importantly, the rate of fulfillment of parenthood increased with increasing time since treatment termination, suggesting that fertility can be regained. Several studies reported that, depending on the gonadotoxicity of the therapy applied and the patients’ age at the time of diagnosis, gonadal function may recover [37–40]. However, these studies also revealed that women are at high risk of premature menopause, which may hamper family planning in women receiving chemotherapy at higher age. In this group of patients, pre-treatment information about the risk of infertility and the available options for fertility preservation are particularly important to enable post-treatment parenthood.

In conclusion, in the ABC study, a large proportion of patients with hematological malignancies received adequate information about the risk of losing fertility, but only a minority decided to preserve it. Women were less likely to be informed and undergo fertility preservation than men. Moreover, childlessness at the time of diagnosis was strongly associated with a desire for post-treatment parenthood, which, however, could only be fulfilled in 30% of cases.

Our observations may gain in importance because first parenthood is increasingly being delayed to higher ages. In the future, more patients are anticipated to express a wish for parenthood after successful therapy of a hematological malignancy. Options to preserve fertility should be considered in all patients and psychosocial support should be offered not only during the time of treatment but also beyond.

## Electronic supplementary material

Below is the link to the electronic supplementary material.

Electronic supplementary material 1 (DOCX 17 kb)

## References

[1] Sant M, Minicozzi P, Mounier M (2014). Survival for haematological malignancies in Europe between 1997 and 2008 by region and age: results of EUROCARE-5, a population-based study. Lancet Oncol.

[2] Zebrack BJ, Casillas J, Nohr L (2004). Fertility issues for young adult survivors of childhood cancer. Psycho-Oncology.

[3] Schover LR, Rybicki LA, Martin BA (1999). Having children after cancer. Cancer.

[4] Schover LR, Brey K, Lichtin A (2002). Knowledge and experience regarding cancer, infertility, and sperm banking in younger male survivors. J Clin Oncol.

[5] Nahata L, Caltabellotta NM, Yeager ND (2018). Fertility perspectives and priorities among male adolescents and young adults in cancer survivorship. Pediatr Blood Cancer.

[6] Wenzel L, Dogan-Ates A, Habbal R (2005). Defining and measuring reproductive concerns of female cancer survivors. J Natl Cancer Inst Monogr.

[7] Cobo A, Meseguer M, Remohi J (2010). Use of cryo-banked oocytes in an ovum donation programme: a prospective, randomized, controlled, clinical trial. Hum Reprod (Oxford, England).

[8] Bizet P, Saias-Magnan J, Jouve E (2012). Sperm cryopreservation before cancer treatment: a 15-year monocentric experience. Reprod Biomed.

[9] Jensen AK, Macklon KT, Fedder J (2017). 86 successful births and 9 ongoing pregnancies worldwide in women transplanted with frozen-thawed ovarian tissue: focus on birth and perinatal outcome in 40 of these children. J Assist Reprod Genet.

[10] Saito K, Suzuki K, Iwasaki A (2005). Sperm cryopreservation before cancer chemotherapy helps in the emotional battle against cancer. Cancer.

[11] Quinn GP, Vadaparampil ST, Gwede CK (2007). Discussion of fertility preservation with newly diagnosed patients: oncologists' views. J Cancer Surviv.

[12] Partridge AH, Gelber S, Peppercorn J (2004). Web-based survey of fertility issues in young women with breast cancer. J Clin Oncol.

[13] Greaves P, Sarker SJ, Chowdhury K (2014). Fertility and sexual function in long-term survivors of haematological malignancy: using patient-reported outcome measures to assess a neglected area of need in the late effects clinic. Br J Haematol.

[14] Quinn GP, Vadaparampil ST, Lee J-H (2009). Physician referral for fertility preservation in oncology patients: a National Study of Practice Behaviors. J Clin Oncol.

[15] Adams E, Hill E, Watson E (2013). Fertility preservation in cancer survivors: a national survey of oncologists' current knowledge, practice and attitudes. Br J Cancer.

[16] Gilbert E, Adams A, Mehanna H (2011). Who should be offered sperm banking for fertility preservation? A survey of UK oncologists and haematologists. Ann Oncol.

[17] Micaux Obol C, Armuand GM, Rodriguez-Wallberg KA (2017). Oncologists and hematologists' perceptions of fertility-related communication - a nationwide survey. Acta Oncologica (Stockholm, Sweden).

[18] Kiserud CE, Fossa A, Holte H (2007). Post-treatment parenthood in Hodgkin's lymphoma survivors. Br J Cancer.

[19] Geue K, Richter D, Schmidt R (2014). The desire for children and fertility issues among young German cancer survivors. J Adolesc Health.

[20] Lee SJ, Schover LR, Partridge AH (2006). American Society of Clinical Oncology recommendations on fertility preservation in cancer patients. J Clin Oncol.

[21] Oktay K, Harvey BE, Partridge AH (2018). Fertility preservation in patients with cancer: ASCO Clinical Practice Guideline Update. J Clin Oncol.

[22] Lambertini M, Del Mastro L, Pescio MC (2016). Cancer and fertility preservation: international recommendations from an expert meeting. BMC Med.

[23] Peccatori FA, Azim HA, Orecchia R, Hoekstra HJ, Pavlidis N, Kesic V (2013). Cancer, pregnancy and fertility: ESMO Clinical Practice Guidelines for diagnosis, treatment and follow-up. Ann Oncol.

[24] Armuand GM, Rodriguez-Wallberg KA, Wettergren L (2012). Sex differences in fertility-related information received by young adult cancer survivors. J Clin Oncol.

[25] Armuand GM, Wettergren L, Rodriguez-Wallberg KA (2015). Women more vulnerable than men when facing risk for treatment-induced infertility: a qualitative study of young adults newly diagnosed with cancer. Acta Oncologica (Stockholm, Sweden).

[26] Niemasik EE, Letourneau J, Dohan D (2012). Patient perceptions of reproductive health counseling at the time of cancer diagnosis: a qualitative study of female California cancer survivors. J Cancer Surviv.

[27] Dolmans MM, Marinescu C, Saussoy P (2010). Reimplantation of cryopreserved ovarian tissue from patients with acute lymphoblastic leukemia is potentially unsafe. Blood.

[28] Meirow D, Hardan I, Dor J (2008). Searching for evidence of disease and malignant cell contamination in ovarian tissue stored from hematologic cancer patients. Hum Reprod.

[29] Mersereau JE, Goodman LR, Deal AM (2013). To preserve or not to preserve: how difficult is the decision about fertility preservation?. Cancer.

[30] Flink DM, Kondapalli LA, Kellar-Guenther Y (2017). Priorities in fertility decisions for reproductive-aged cancer patients: fertility attitudes and cancer treatment study. J Adolesc Young Adult Oncol.

[31] Armuand GM, Wettergren L, Rodriguez-Wallberg KA (2014). Desire for children, difficulties achieving a pregnancy, and infertility distress 3 to 7 years after cancer diagnosis. Support Care Cancer.

[32] van der Kaaij MA, Heutte N, Meijnders P (2012). Parenthood in survivors of Hodgkin lymphoma: an EORTC-GELA general population case-control study. J Clin Oncol.

[33] Meissner J, Tichy D, Dietrich S (2014). Parenthood in long-term survivors after CHOP with or without etoposide treatment for aggressive lymphoma. Br J Haematol.

[34] Gerstl B, Sullivan E, Koch J (2019). Reproductive outcomes following a stem cell transplant for a haematological malignancy in female cancer survivors: a systematic review and meta-analysis. Support Care Cancer.

[35] Chiodi S, Spinelli S, Bruzzi P (2016). Menstrual patterns, fertility and main pregnancy outcomes after allogeneic haematopoietic stem cell transplantation. J Obstet Gynaecol.

[36] Borgmann-Staudt A, Rendtorff R, Reinmuth S (2012). Fertility after allogeneic haematopoietic stem cell transplantation in childhood and adolescence. Bone Marrow Transplant.

[37] Gharwan H, Lai C, Grant C (2016). Female fertility following dose-adjusted EPOCH-R chemotherapy in primary mediastinal B-cell lymphomas. Leuk Lymph.

[38] Elis A, Tevet A, Yerushalmi R (2006). Fertility status among women treated for aggressive non-Hodgkin's lymphoma. Leuk Lymph.

[39] Behringer K, Mueller H, Goergen H (2013). Gonadal function and fertility in survivors after Hodgkin lymphoma treatment within the German Hodgkin Study Group HD13 to HD15 trials. J Clin Oncol.

[40] Anderson RA, Remedios R, Kirkwood AA (2018). Determinants of ovarian function after response-adapted therapy in patients with advanced Hodgkin's lymphoma (RATHL): a secondary analysis of a randomised phase 3 trial. Lancet Oncol.

