# Supplementary material for: Fertility preservation and fulfillment of parenthood after treatment of hematological malignancies: results from the ‘Aftercare in Blood Cancer Survivors’ (ABC) study
Source: Int J Clin Oncol. 2020 Mar 5;25(6):1187–94. doi: 10.1007/s10147-020-01639-4 (PMC7261262; doi:10.1007/s10147-020-01639-4)
Supplement: Supplementary file 1 — Electronic supplementary material 1 (DOCX 17 kb) [file 10147_2020_1639_MOESM1_ESM.docx]

**Online Supplementary Material**

**Fertility preservation and fulfillment of parenthood after treatment of hematological malignancies: Results from the ‘Aftercare in Blood Cancer Survivors’ (ABC) study**

Christine Schmitz^1^, Julia Baum^1^, Hildegard Lax^2^, Nils Lehmann^2^, Tanja Gromke^3^, Dietrich W. Beelen^3^, K.-H. Jöckel^2^, and Ulrich Dührsen^1^

Fertility-related questions

1. Before start of cancer-specific treatment, have you been informed,
   that therapy may negatively affect your fertility?


   ( ) Yes.
   ( ) No, although I have been at an age, where fertility mattered to me.
   ( ) No, since I was at an age, where fertility did not matter to me any longer.
   ( ) The question does not apply to me, since my disease did not require
    treatment so far.
2. Have you decided to undergo fertility preservation?


   ( ) Yes, I did cryopreserve my sperm.
   ( ) Yes, I did cryopreserve oocytes or ovarian tissue
   ( ) Yes, I used medications to supress my menstruation cycle (e.g. GnrH analoga).
   ( ) No, I did not know, that any of the above mentioned methods exist.
   ( ) No, because need for treatment was too urgent to undergo fertility preservation.
   ( ) No, the possibility of having children at a later point in time was
    not important to me at the time when the treatment started.
   ( ) The question does not apply to me, since I was at an age, where
    fertility did not matter to me any longer.
   ( ) The question does not apply to me, since my disease did not require
    treatment so far.
3. Have you ever wanted to have children after the time of diagnosis (in
   cases of untreated diseases), after the end of therapy or under
   current medication?
   (multiple answers are possible)


   ( ) Yes.
   ( ) No, my family planning was terminated.
   ( ) No, because of fear against diesease relapse or progression.
   ( ) No, because of the fear against cancer disease of the child.
   ( ) No, because of financial concerns.
   ( ) The question does not apply to me, since I was at an age, where
    fertility did not matter to me any longer
   ( ) Other, which is ________________________
4. Could you continue your desire to have a child after time of diagnosis
   (in cases of untreated diseases), after end of therapy, or under
   current medication?


   ( ) Yes.
   ( ) No, although I tried to.
   ( ) No, because I had no partner.
   ( ) I did not have a desire to conceive, since I was at an age, where
    fertility did not matter to me any longer
   ( ) Other, which is _____________________
5. If you had a desire to conceive, which could not be fulfilled immediately: Which examinations have been performed, to explore the
   reason  for infertility?
   (multiple answers are possible)

( ) No examinations
( ) Consultation of a gynecologist
( ) Consultation of an urologist
( ) Consultation of a reproduction clinic
( ) Hormone check-up
( ) Spermiogram
( ) The question does not apply to me, since I was at an age, where
 fertility did not matter to me any longer
( ) Other, which is _____________________

1. How could you fulfill your wish for a child after time of diagnosis
   (in cases of untreated diseases), after end of therapy, or under
   current medication?


   ( ) Natural conception
   ( ) I used assisted reproduction tecniques, which are ______________
   ( ) I used my cryopreserved sperms to conceive a child.
   ( ) We adopted a child.
   ( ) I did not have a desire for a child.
   ( ) The question does not apply to me, since I was at an age, where
    fertility did not matter to me any longer
   ( ) Other, which is _____________________
2. Can you please give us information, how many children you got before and after cancer-specific treatment

|  | Number of children, that were born/adopted before cancer diagnosis | Number of children, that were born/adopted after cancer diagnosis |
| --- | --- | --- |
| Natural conceived children |  |  |
| Children that were conceived using assisted reproduction teqniques |  |  |
| Adopted children |  |  |
|  |  |  |

1. How much do fertility-related problems affect your life?

( ) This question does not apply to me, since I have no fertility-related problems that are due to my cancer diagnosis or its treatment.

( ) Please circle the number, that represents your burden:

0 1 2 3 4 5 6 7 8 9 10

No effect very strong
